# [Retracted] Proteasome inhibitor sensitizes oral squamous cell carcinoma cells to TRAIL‑mediated apoptosis

**DOI:** 10.3892/or.2023.8519

**Published:** 2023-03-06

**Authors:** Sayaka Yoshiba, Masayasu Iwase, Sayaka Kurihara, Makiko Uchida, Yuji Kurihara, Hitoshi Watanabe, Satoru Shintani

Oncol Rep 25: 645–652, 2011; DOI: 10.3892/or.2010.1127

Following the publication of this paper, it was drawn to the Editor's attention by a concerned reader that, in Fig. 4 on p. 650, the same β-actin bands had apparently been used to show the experimental effects of the proteasome inhibitor MG-132 on c-FLIP in HSC-2 cells in Fig. 4A, and the effects of MG-132 on IAPs in HSC-3 cells in Fig. 4B. In addition, for the fourth lane in the gel showing the effects of MG-132 on c-FLIP in HSC-3 cells, this should have been labelled as ‘+MG-132 / +TRAIL’ (not as ‘-/-’). Upon contacting the authors in relation to this matter, they could only admit that errors had been made in the preparation of the figure; moreover, they no longer had access to the original data owing to the time that has elapsed since the publication of the paper, and it would be impossible for them to now repeat this experiment. After having considered this matter and in conjunction with a request made by the authors, the Editor of *Oncology Reports* has decided that this paper should be retracted from the publication. Both the Editor and the authors apologize to the readership for any inconvenience caused.

